# Taking cues from ecological and evolutionary theories to expand the landscape of disgust

**DOI:** 10.1098/rspb.2024.1919

**Published:** 2024-12-04

**Authors:** A. E. Love, A. M. Heckley, Q. M. R. Webber

**Affiliations:** ^1^ Department of Integrative Biology, University of Guelph, Guelph, Ontario, Canada; ^2^ Department of Biology and the Redpath Museum, McGill University, Montreal, Quebec, Canada

**Keywords:** coevolution, disease ecology, habitat selection, host–parasite dynamics, natural selection, parasite avoidance

## Abstract

Behavioural avoidance of parasites in the environment generates what is known as the ‘landscape of disgust’ (analogous to the predator-induced ‘landscape of fear’). Despite the potential for improving our inference of host–parasite dynamics, three limitations of the landscape of disgust restrict the insight that is gained from current research: (i) many host–parasite systems will not be appropriate for invoking the landscape of disgust framework; (ii) existing research has primarily focused on immediate choices made by hosts on small scales, limiting predictive power, generalizability, and the value of the insight obtained; and (iii) relevant ecological and evolutionary theory has yet to be integrated into the framework, challenging our ability to interpret the landscape of disgust within the context of most host–parasite systems. In this review, we explore the specific requirements for implementing a landscape of disgust framework in empirical systems. We also propose greater integration of habitat selection and evolutionary theories, aiming to generate novel insight, by exploring how the landscape of disgust varies within and across generations, presenting opportunities for future research. Despite interest in the impacts of parasitism on animal movement and behaviour, many unanswered questions remain.

## 1. Introduction

Individual organisms interpret cues from their environment to evaluate risks and inform decisions to increase fitness. Animal behaviour can change based on perceived associations between a given cue and potential fitness costs or benefits, in time leading to possible behavioural adaptation and predictable responses [1,2]. When cues are associated with the risk of infection with micro- or macro-parasites (hereafter ‘parasites’ [3]), the perception of cues can result in feelings akin to the human feeling of disgust [4–6] and can elicit behavioural avoidance of infectious agents [5–12]. The distribution of cues in an environment and the resulting spatial distribution of organisms has been termed the ‘landscape of disgust’ [4], analogous to the predator-induced ‘landscape of fear’ in which organisms respond to predator-associated cues (*sensu* [13]). By describing the cues that hosts encounter, and thus may interpret and respond to, the landscape of disgust is a tangible and interactive property of host–parasite systems. The landscape of disgust framework has motivated increasing research on the ecological impacts of parasitism in nature [4,14,15], and in particular, non-consumptive effects (i.e. impacts to hosts beyond direct consumption, including changes to behavioural and developmental traits, among other traits [16–18]).

Despite the potential value of the landscape of disgust framework, three key limitations restrict the applicability of the landscape of disgust in natural systems. First, in many natural host–parasite systems, the landscape of disgust does not exist because potential hosts will not always behaviourally avoid infection risks [5,6]. Although parasite infection risks are ubiquitous in nature, the costs of parasitism vary widely—ranging from infections with limited fitness impacts to infections that cause mass mortality as they spread throughout populations [19]. In addition to direct negative effects on survival, parasites can have also indirect fitness effects by increasing the variability of reproductive success [20]. Application of the landscape of disgust relies on the existence of cues and the ability of potential hosts to recognize and respond to those cues [1,8], which would not be present for many asymptomatic infections. For infections that have strong fitness impacts, and therefore selection for preventing infection, detectable cues may allow for selection of behavioural avoidance. Infections with no detectable cues may result in selection for alternative strategies to prevent fitness costs such as increased resistance (the ability to limit or prevent infection after contact with a parasite) or tolerance (the ability to reduce fitness consequences of infection) [21]. Second, in systems where the landscape of disgust does exist, empirical research has focused on small spatiotemporal scales. Although small-scale studies remain critical for improving mechanistic understanding of disgust, limited research at larger scales results in a knowledge gap about how parasite avoidance scales up to landscapes. Increasing the spatiotemporal scales of the landscape of disgust is indeed critical for increasing the value of the framework, although complexities introduced at higher scales may seem likely to create challenges for interpreting the findings of empirical work. We suspect the first two limitations are rooted in the basis of our third argument: the current landscape of disgust framework is limited by a lack of integration of relevant ecological theory (e.g. habitat selection theory [22]) and evolutionary theory (e.g. foundational evolutionary forces driving adaptation [23]). Incorporating these broad bodies of literature will enhance robustness and ease interpretability for those seeking to investigate the landscape of disgust in natural settings and at larger scales.

**Figure 1.**
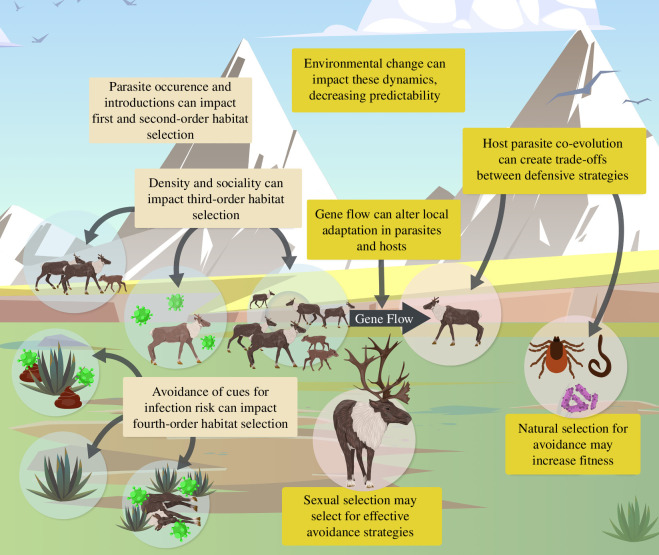
Factors affecting the formation and demonstration of the landscape of disgust. Here, we illustrate a hypothetical system to demonstrate different factors that affect the landscape of disgust both within (tan boxes on the left) and across (yellow boxes on the right) generations. These factors include principles from habitat selection theory operating at varying spatial scales that could be impacted by the detection of risk and ecological context, along with evolutionary principles such as selection for traits associated with the detection and avoidance of parasites, or gene flow or genetic drift impacting genetic variation for selection. This is not an exhaustive representation of factors impacting the landscape of disgust but demonstrates several factors that should be considered when interpreting avoidance behaviour and making predictions in natural systems. Illustrated by Juan Aristizabal.

Our review aims to illustrate how critical insight could be gained by expanding the landscape of disgust interpretation to include habitat selection theory (§2; figure 1) and evolutionary theory (§3; figure 1). To provide context for this review, we provide background knowledge of the landscape of disgust (box 1), and a visual aid to show how aspects of the landscape of disgust could change within and across generations (box 2). We finally provide examples of outstanding questions and suggestions on how to empirically approach researching these topics to expand our understanding of the landscape of disgust (§4; box 3).

Box 1: **Describing the landscape of disgust**

The landscape of disgust represents behavioural responses to parasite infection risks [4]. Researchers have emphasized the role of host anti-parasite behaviours for decades [1,24], and the landscape of disgust provides a framework to renew previous lines of research, inspire new approaches to classic questions, and concentrate research efforts on the investigation of non-consumptive effects of parasitism.

Host–parasite dynamics influencing the landscape of disgust can vary between species, populations, and individuals within populations. For a landscape of disgust to exist and impact host behaviour, there are at least three prerequisite environmental, physiological and evolutionary factors required. First, a cue—which can be visual, auditory, olfactory or mechanosensory—must be produced either directly by a parasite, the infected host or something associated with the risk of infection [6]. For example, the avoidance of faeces which may or may not be infested with parasites. Second, hosts must have the capacity to detect cues; the physiological mechanisms to interpret and recognize the cue are required for the cue to be perceived by the host within the landscape of disgust (e.g. detection of chemical cues in mice [25]). Finally, parasitism must be costly enough to impact potential host behaviour, and this change must prevent or reduce infection resulting in a fitness benefit to the host [5,6]. Both cue detection and response behaviour could be learned [26–28] or adaptive if sufficient time and genetic variation are present to evolve a connection between the cue and response [29]. Selection can act on phenotypes involved in different stages of infection, including recognizing parasite cues, and responding to infection [30]. In natural systems, these prerequisites for the landscape of disgust can take on many forms; any breakdown in the connection between the cue production, perception and response will prevent a quantifiable landscape of disgust from forming.

Box 2: **Visualizing landscape of disgust within and across generations**

**Figure B1.**
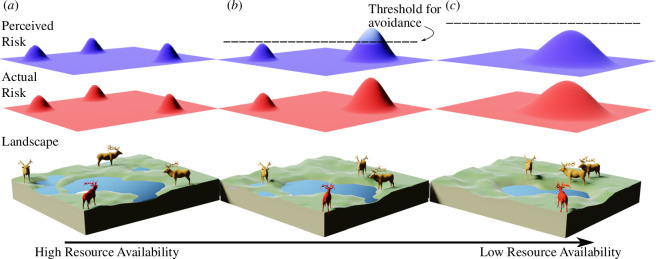
Visual depiction of a hypothetical change to the landscape of disgust that can occur across seasons, within a generation of a host. A critical resource (water) becomes increasingly scarce from wet to dry seasons, increasing density of conspecifics (animals per unit area) around water sources that could harbour infection through time. The geographic landscape and conspecific locations (lower layer), the actual risk of infection (middle layer), and the perceived risk of infection (upper layer) are denoted as layers in each panel. From (*a*) to (*b*), as the resource becomes less available, individuals will choose lower-density areas that present less risk but still provide access to the resource (where perceived risk is below threshold in the upper layer). Between (*b*) and (*c*), the threshold of avoidance (upper layer) becomes higher as the resource becomes increasingly scarce. In this example, individuals will choose to increase their risks of infection (middle layer) by aggregating to gain access to remaining water resources (lower layer).

**Figure B2.**
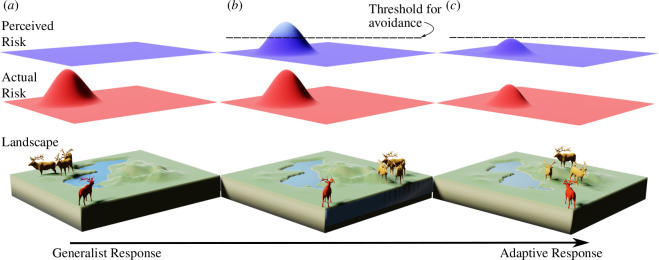
Visual depiction of a hypothetical change to the landscape of disgust over generations. A novel environmentally transmitted parasite is introduced to a landscape via a contaminated waterbody that represents the environmental reservoir; the introduction of this parasite drives adaptive changes in the host population over generations. The geographic landscape and conspecific locations (lower layer), the actual risk of infection (middle layer), and the perceived risk of infection (upper layer) are denoted as layers in each panel. Between (*a*) and (*b*), the host population evolves to associate the parasite cue with the environmental reservoir (there is a novel perceived infection risk) (upper layer). Between (*b*) and (*c*), the host population has evolved to become more tolerant to infection by that parasite (reduction in the magnitude of actual risk in the middle layer), reducing the perceived cost of infection; notably, there is a shift in the magnitude of perceived infection risk such that the perceived risk is now lower than the actionable avoidance threshold.

We visualize the landscape of disgust as the summation of any ‘perceivable’ parasite infection risks within an individual’s spatial perception, relative to the ‘active’ infection risks (figures B1 and B2). We primarily discuss and interpret the landscape of disgust as a two-dimensional plane, as this reflects the movement of most terrestrial individuals, although in some systems a third axis of movement is available to avoid parasite infection, such as in aquatic systems [12], or for aerial or arboreal animals that can use vertical movement to avoid transmission (e.g. [31]). Because not all parasites can be detected, and not all perceived risks warrant a response [5,6], we visualize a biologically relevant threshold for infection risk that determines whether an avoidance response occurs at all (represented by the horizontal line overlapping perceived risks in figures B1 and B2), which parallels the ‘cost–benefit’ structure used in other parasite avoidance and disgust reviews [5,6,11]. An avoidance threshold can be affected by the individual state or past experiences, such as an individual’s body condition or hunger levels [32] and prior or current infection status [33,34]. Although we illustrate individual avoidance as binary in the context of this threshold, the strength of an avoidance response can vary such that individuals may moderate their foraging behaviour to decrease exposure (weaker response) or leave the area to seek lower-risk habitat (stronger response). We predict the avoidance response to be correlated with the size of the perceived risk (represented as height of peak in figures B1 and B2). The risk of parasite infection also often exists as a gradient, and variation in parasite risk can occasionally be detected (e.g. [35,36]); this variation in risk is represented with simple peaks here but in natural systems there will be variation in the gradient around the cue as well [37]. In visualizing the landscape of disgust across two time scales—within a generation (figure B1) and across generations (figure B2)—we provide a visual aid to help the reader understand how ecological and evolutionary theory fit into, and would expand, the existing framework.

Box 3: **Tools for measuring and interpreting the landscape of disgust in nature.**

(i) Mapping parasites on the landscape: To predict both the benefits of avoidance and where avoidance should occur, quantifying the parasites that actually exist in a given environment provides important context. It is possible to map parasite distributions when they are inside hosts or outside hosts. Species distribution models (SDMs) [38] and resource selection functions (RSFs) [39] are useful tools for estimating organismal distribution through space and time. One way to estimate parasites without intermediate or external stages (e.g. viruses) is to develop SDMs or RSFs for infected hosts through space and time (e.g. [40]). For hosts with intermediate or external stages (e.g. nematodes or ectoparasites), we propose a multi-step approach, similar to the joint-SDM approach [41], where SDMs or RSFs are developed for definitive hosts, intermediate hosts, and/or for the parasites themselves while they are outside of the host.

(ii) Agent-based modelling of the landscape of disgust: Agent-based models are spatially explicit individual-level models. The emphasis on the individual level (the agent) provides an excellent opportunity to explore decision making and trade-offs when perceiving risks. These models are used to investigate movement and processes from individual-level physiological processes (e.g. energetics [42]) up to ecosystem-level processes (e.g. landscape heterogeneity [43]). Researchers could model a landscape of disgust with agents that are a source of risk (i.e. social transmission), or that create risks (e.g. faecal deposition), tracking how individuals who perceive these risks avoid them and how this could impact the system at varying levels (e.g. disease dynamics or nutrient cycling).

(iii) Tracking the landscape of disgust across generations: To estimate the strength and direction (e.g. stabilizing, disruptive) of selection in the context of the landscape of disgust, a researcher could measure any given trait (e.g. avoidance behaviour, resistance or tolerance) and a fitness proxy (e.g. number of offspring that survive to reproductive age, number of offspring produced) [23]. If the traits of interest are heritable (which can be estimated in different ways, such as the traditional method of correlating offspring to parental phenotypes [44]), researchers can gain insight into whether selection on a trait (or correlated traits) will lead to evolutionary change in the landscape of disgust [45,46].

## 2. Landscape of disgust within a generation

Habitat is a location in environmental space, defined by a set of conditions (e.g. temperature), resources (e.g. food) and risks (e.g. predators or parasites) [47], with habitat selection being the process through which animals differentially use habitats relative to their availability at a given population density to maximize fitness [48]. Habitat selection theory provides a foundation for assessing how the landscape of disgust will be shaped by factors that vary within the lifetime of an individual. In this section, we first discuss how the landscape of disgust will be shaped by the dynamic effects of parasite transmission, host density and host sociality (§2a). Next, we consider ways to increase the spatial and temporal scales at which the landscape of disgust is assessed within a generation (§2b).

### (a) Density dependence and sociality

Habitat selection, by definition, is density-dependent [49,50]. Following the ideal free distribution and density-dependent habitat selection theory, animals should select habitat to maximize fitness relative to the availability of habitat, resulting in varying population densities among habitats in proportion to the fitness value of each habitat [48,51]. Density-dependent habitat selection theory therefore provides a null expectation for how animals select habitat within the context of the conditions, resources and risks that make up their environment. However, the conceptual link between density-dependent habitat selection and parasitism is lacking; an absence which is striking given that density is often associated with parasite transmission risks [52,53].

An increase in host population density is predicted to result in more social contacts and therefore higher parasite transmission, and this effect has been observed in several meta-analyses (e.g. [54,55]). However, transmission does not always increase with increasing host density owing to the encounter-dilution effect [56], or because of increased (natural) selection for individual-level avoidance behaviours (e.g. [57]). Associations between host density and parasite infection risk can change over time (e.g. [58]). For example, increased density surrounding waterholes in dry seasons substantially increases the prevalence of oral-faecal parasites, causing a tight association between seasonality and parasite prevalence [59] (figure B1). Notably, it is not solely host conspecific density that can impact risk, but heterospecific host density can also impact risk [60]. To provide important context to the landscape of disgust, both host social behaviour and density-dependent habitat selection must be incorporated into estimates of the landscape of disgust (figure 1).

The landscape of disgust does not only exist for uninfected individuals avoiding infectious agents. Infected individuals with clinical symptoms are also a part of the landscape of disgust (figure B1*a*) and their behaviours affect how uninfected and infected conspecifics interact and the extent to which they can avoid infection risks in the environment. For instance, parasites can impact social behaviour to facilitate further infection (e.g. increased shoaling in infected fish [61]) or alternatively, parasite infection can maintain social behaviour even as host movement and habitat selection change [62]. Conversely, infected individuals may not be accepted into social groups (e.g. guppy, *Poecilia reticulata*, shoals avoid infected conspecifics) [63], reducing the risk for all individuals in the group and altering the structure of the landscape of disgust. Indeed, whether an animal is solitary or social can impact the behavioural defences employed against parasites, including avoidance [10]. In some cases, infected conspecifics with infection-associated pathologies are not avoided. For example, eastern water dragons (*Intellagama lesueurii*) do not avoid conspecifics infected with a lesion-causing fungus unless the severity of the infection is severe, presumably because the benefits of sociality outweigh the costs of most infections [35].

Social behaviour contributes to the spatial structuring of populations [64], which in turn affects the placement of one type of infection risk (i.e. direct transmission from conspecifics) on the landscape of disgust. Although the infection risk of directly transmitted parasites (i.e. parasites that require direct contact between hosts for transmission [65]) is generally higher in large social groups [55,66], social behaviours, such as allogrooming and social learning of parasite cues, can offset costs of living in large groups [26,67]. Further, animals can modify their movement to reduce infection risks for themselves or group mates. For instance, primates are thought to cycle through sleeping groves and defecate in specific areas to reduce parasite transmission [68,69], bats avoid recolonization of recently used roosts with potential for infection [70], and badgers (*Meles meles*) moderate sett usage based on infection risk [57]. The landscape of disgust can also be shaped by social hosts mitigating risk by controlling where they generate risks (e.g. latrines or defecation behaviour). Taken together, the interactive effects of host sociality, habitat selection, density and parasitism are multifaceted and can contribute to a dynamic landscape of disgust within the lifetime of an individual or group of individuals. It is well known that sociality and density operate on various biological scales [64], suggesting that the interactions with the landscape of disgust will vary from small to larger scales.

### (b) Expanding spatiotemporal scales

Habitat selection theory proposes that habitat decisions are hierarchical [71,72]; animals select habitats first at larger spatial scales and then make smaller scale decisions within habitats. Habitat selection is considered at four scales: first order (the geographical area used by a species), second order (the home range of an individual or group), third order (the resource selection decisions made by individuals within their home range) and fourth order (an individual’s immediate decision making, often related to foraging decisions) [73]. Most landscape of disgust research focuses on third- and fourth-order scales. Intake maximization is the most heavily studied driver of habitat selection in the context of landscape of disgust (e.g. [74–76]). Some food sources, such as carcasses and faeces, are high risk for a host to acquire parasites. As a result, they should be avoided behaviourally while foraging at the fourth order of habitat selection [33,77]. Quantifying behavioural avoidance at the fourth order could be done using cafeteria-style experiments to measure ‘give up density’ (a metric used to indicate when an animal quits harvesting from a patch) [13]. Within the third and fourth orders of habitat selection, we would expect individuals to typically avoid areas or resources where parasite-associated cues are perceived (e.g. [78,79], figure 1). However, in natural systems, risk-free habitats may not exist, leading to trade-offs in habitat selection. For example, individuals typically favour taking parasite risks over predation risks [80], though not always (e.g. additive avoidance responses [81]).

Connecting ecological processes across the orders of habitat selection has become a hallmark of empirical and theoretical habitat selection studies. Because the landscape of disgust should correlate with infection risk for a given parasite, variation in this risk across large spatiotemporal scales might predict avoidance behaviour and generate broad-scale patterns in finer scale avoidance behaviour [82]. Broad-scale variation in parasite infection risks could emerge owing to climatic gradients like temperature or precipitation that can predict parasite prevalence at population or species levels [83]. Temperature can also affect host immune function [84], potentially changing the effectiveness and benefits of avoidance behaviour at the third and fourth orders of habitat selection. At the third order of habitat selection, some animals can use behaviours such as migration to seasonally avoid infested habitats and reduce parasite prevalence as they traverse diverse climatic conditions [85]. These effects on avoidance could be most dramatic in unfavourable environments, such as at species range limits where host condition may be lower [86], affecting trade-off dynamics between avoiding fitness costs associated with parasite infection and satisfying other needs, such as foraging or mating. Drivers of broad-scale patterns could also depend on community composition. Diversity varies at large spatial scales, with the most diverse communities often occurring closer to the equator [87], potentially resulting in a reduction in avoidance behaviour where the dilution effect is observed (i.e. increased diversity is associated with decreased parasite prevalence; [88]). Considering large-scale environmental and ecological processes should strengthen inferences when predicting the strength and variation in the landscape of disgust as it spans across these different orders of habitat selection.

In the context of avoidance behaviour, this relationship between different orders of habitat selection is likely akin to a feedback loop. The presence or efficacy of avoidance at one order may impact the need for avoidance at higher or lower orders. Although we expect animals to place their home ranges to minimize infection risk [89], home ranges may still include areas with higher risk (third order [90]), in which case finer scale avoidance is an important adaptive behaviour (fourth order [91]). Another aspect that may affect the relevance of avoidance behaviours is the predictability of risk [92]. In areas with high spatial and temporal predictability in infection risk, third- and fourth- order avoidance behaviours should be most effective as individuals can reliably change their foraging behaviour and habitat selection to prevent risk exposure. In contrast, when the predictability of risk in space or time is low the efficacy of fine-scale avoidance is lower and therefore it may be more adaptive for individuals to invest in other anti-parasite defences such as resistance or tolerance. Indeed, the predictability of risk could impact the trade-offs for selecting a habitat that has associated infection risk. If there is a high fitness cost to infection and no potential for fine-scale avoidance at the fourth order of habitat selection then we expect avoidance at the third- or second-order habitat selection decreasing exposure to risky areas [93].

We suggest the consideration of avoidance in habitat selection behaviour at different scales should be a priority for future empirical work. At the first and second orders of selection, biogeographic patterns of parasite avoidance behaviour can provide insight into historical avoidance or the factors driving selection for these traits at smaller scales. The process of scaling up the landscape of disgust from the fourth order to the first order of habitat selection relies on the integration of macroecological principles with existing knowledge and theory [82]. Additional inference could be gained by correlating the propensity for fine-scale avoidance behaviours with an individual’s patterns of habitat selection (third order). Similarly, estimating individual, population or species-level niche partitioning within the context of behavioural avoidance of parasites could shed light on the integration of avoidance across scales. Researchers could use meta-analyses that combine small-scale studies of avoidance behaviour among populations or species at different spatial scales (with carefully selected moderators to untangle sources of variation in behavioural responses). One caveat to this approach is that it requires sufficient data on avoidance, and therefore might not be immediately feasible and awaits future work. Broader scale research is necessary given that small-scale processes, although providing important mechanistic insight, rarely scale linearly and emergent properties at the higher organizational levels require broad-scale investigation to be identified [82].

## 3. Landscape of disgust across generations

Within an animal’s lifetime, the landscape of disgust can be static or dynamic depending on how various mechanisms develop or continue to develop via evolutionary processes (figure 1). Evolution generating variation in the landscape of disgust could be reflected in many ways, including the mechanisms that hosts use to recognize and avoid parasites, their ability to resist or tolerate infections by certain parasite species (‘ghosts of parasitism past’ [94]), or through non-behavioural avoidance (e.g. morphological adaptations [11,95]). Variation will affect all three components of the landscape of disgust framework: actual infection risks, perceived infection risks and the threshold for avoidance behaviour. Not all variation in the landscape of disgust is a product of evolution; plasticity could underlie some phenotypic variation, although plasticity can also be a product of evolutionary forces [96]. Below, we discuss how the landscape of disgust will be shaped by evolutionary change in the hosts (§3a), focusing on evolutionary processes that drive those changes (i.e. natural selection, sexual selection, gene flow and drift). Next, we discuss how the landscape of disgust will be shaped by evolutionary changes in the parasites, often in response to host evolution (§3b).

### (a) Host evolution

The most apparent process by which evolution could generate change in the landscape of disgust over time is natural selection. When infection has negative fitness consequences, and where heritable variation exists in the traits that affect fitness, natural selection should drive adaptation to improve avoiding, resisting or tolerating infection [7,11]. In the landscape of disgust, natural selection could improve cue detection (adding resolution to the landscape of disgust; see figure B2*a,b*) and increase avoidance of risks (how an individual reacts to the landscape of disgust it perceives). Importantly, cue detection and avoidance are likely under correlated selection, as these traits go hand in hand [97]. Detection and avoidance also presumably correlate with resistance and tolerance [6,11]. Populations with high resistance or tolerance may not have as strong selection for avoidance behaviours, as they handle infection with a different strategy [7,27,98]; in which case, high resistance or tolerance strategies could translate to a higher threshold for parasite avoidance (figure B2*c*) or a lower perceived risk (e.g. decreased detection of parasite cues). For instance, house finches (*Carpodacus mexicanus*) that have stronger behavioural avoidance responses invest less in immune defences [99]. Raccoon (*Procyon lotor*) latrines tend to have a high prevalence of raccoon roundworm (*Baylisascaris procyonis*), and tolerant species (e.g. raccoons and rats) use latrines frequently, whereas intolerant species (e.g. birds and small mammals) avoid them [77]. Although these ‘strategies’ (cue detection versus altered avoidance threshold versus resistance and tolerance) may be difficult to tease apart empirically, modelling provides an avenue that could attempt to investigate the independent effects of these strategies (box 3). For instance, theory suggests that the evolutionary dynamics leading to behavioural defences can differ from resistance or tolerance depending on avoidance behaviour type and cost of infection [100]. Researchers could also conduct comparative analyses among populations or species that occupy different environments that might be selected for different cue detection methods, as detection mechanisms are likely highly associated with the organism’s ecology and the environment they inhabit [6].

Sexual selection could also generate variation in host−parasite defences. For instance, more vibrant or ornamented individuals are typically hypothesized to be preferred by the choosier sex because they are more resistant to parasites and hence can afford to produce energetically costly ornamentations [101]. When the choosier sex selects mates that are more resistant, tolerant or best at avoiding infection, variation in parasite avoidance could arise, leading to variation in the landscape of disgust if subsequent generations inherit these anti-parasite defences. Increased anti-parasite defences owing to sexual selection could present in the landscape of disgust similar to the outcomes of natural selection described above (e.g. high resistance or tolerance could result in decreased avoidance). However, natural selection could also remove individuals from populations that are the most resistant, tolerant or least effective at avoiding infection if the individuals that cope best with parasite infections (i.e. that are more conspicuous) are also more likely to be predated upon (e.g. [102]). The balance (‘trade-off’) between these two selective pressures will likely impact the landscape of disgust, owing to selection acting differently on host behaviour. In empirical studies, this expectation that healthier and more conspicuous individuals will be preferred by the choosier sex is often not met [103]. Many hypotheses exist to explain the lack of expected trade-offs, including that the association will be shaped by characteristics of the host or parasite (e.g. [104]), that the association is not being investigated at the appropriate scale of inference (e.g. [103]), or that other sources of selection in the environment could be ‘confounding’ the expected association (e.g. [105]). In other words, the association can be context-dependent and is likely to be variable within and among populations but could nevertheless help to shape the landscape of disgust over time.

Natural and sexual selection are not the only mechanisms that can generate evolutionary change. Some host populations are more susceptible to genetic drift (e.g. if they are small and isolated [106]), and the associated randomness could create challenges for predicting parasite avoidance as it relates to the landscape of disgust. In systems where behavioural defences are particularly effective at reducing infection, it has even been suggested that immune-based responses could be lost through drift [7]. Additionally, in connected populations where individuals disperse, gene flow could affect host–parasite dynamics [107] and the landscape of disgust over time. As an example, the introduction of individuals from a different population that have not coevolved with a given parasite could reduce the extent to which the resident population is locally adapted to those parasites, which could impact selection [108]. Specifically, gene flow could swamp out evolved defence mechanisms, such as cue recognition or avoidance behaviours, essentially ‘resetting’ the landscape of disgust. In such cases, decreases in the accuracy of risk perception (upper layer in figure B2) or avoidance behaviours may be observed. Alternatively, the introduction of genetic variation could shift the landscape of disgust by facilitating adaptation and the potential for more effective anti-parasite responses to evolve.

### (b) Parasite (co)evolution

Evolutionary processes affect the landscape of disgust over longer time scales as frequency-dependence or ‘arms race’ dynamics play out in host–parasite systems [11]. While the host is ‘winning’ the arms race, the cost of infection may be reduced due to shifts in behaviour, resistance and tolerance; however, similar evolutionary processes also occur for parasites, and selection can drive variation in adaptive parasite traits (e.g. [109]). The strength of selection acting on parasites is highly dependent on host defences. If host populations evolve increased tolerance, parasites may not suffer substantially reduced fitness, and so natural selection acting on the parasites will be weak [110]. In contrast, if host populations evolve increased resistance, parasite fitness will decrease, and there will be strong natural selection acting on parasites. Likewise, if host avoidance strategies are successful at reducing infection, then parasite populations could decrease, reducing the strength of selection acting on hosts but increasing the strength of selection on the parasites to rapidly adapt in response [11]. When selection on parasites is strong, the parasites may evolve less noticeable cues, or shifts may occur in the presentation of disease caused by the parasite, limiting the efficacy of avoidance behaviours for reducing infection risks. For example, for some viruses, such as SARS-CoV-2, infectiousness is highest prior to the onset of symptoms [111] (impacting the upper layer in figure B2). In this sense, parasites can adapt in response to host evolution to successfully infect despite their presence in the landscape of disgust (the former scenario) or can avoid entering the landscape of disgust in the first place (the latter scenario; at least for the pre-symptomatic period).

In many cases, humans alter the movement of animal hosts and parasites, increasing interactions between hosts and parasites that have no, or weak, coevolutionary histories, which can have devastating impacts on host populations [112]. The movement of hosts or parasites could introduce novel parasite species to host populations, or familiar parasite species (i.e. a species that the host has coevolved with) from genetically distinct populations that the host has not coevolved with [113]. Such scenarios are hypothesized to explain the success of some invasive species (i.e. the novel weapons hypothesis [114]). For example, when the American grey squirrel (*Sciuris carolinensis*) was introduced to Europe it also introduced a parapox virus, contributing to the grey squirrels' ability to outcompete Eurasian red squirrels (*S. vulgaris*), a host that had no previous exposure to the virus [115]. Scenarios of novel host–parasite interactions highlight the importance that evolutionary histories or genetic backgrounds can have in host–parasite dynamics. The importance of shared evolutionary histories in shaping the landscape of disgust (affecting actual risks, perceived risks and host responses to those risks) is a promising avenue for future work, both in natural contexts and with increasing anthropogenic impacts to host–parasite interactions.

## 4. Conclusions and future directions

In this review, we emphasize the value gained by incorporating habitat selection and evolutionary theories into the landscape of disgust framework. There are many practical ways to integrate concepts from ecological and evolutionary theory into the landscape of disgust that could be leveraged in future work (box 3). We recognize that determining the level of information required to map actual and perceived infection risks in natural systems may be difficult. The work required to quantify and map or predict the infection risks a host may encounter, or the evolutionary processes acting on hosts and parasites, poses several logistical barriers including difficulty detecting parasites, the time required to collect data, and potential cost or technological barriers. One solution is to use simulation tools such as agent-based modelling [116] to investigate how the landscape of disgust changes and how it can impact other aspects of natural systems to inform future empirical work (box 3).

Our review highlights that the landscape of disgust remains in its infancy; without an understanding of how the landscape of disgust changes within and across generations, we cannot fully comprehend how parasite infection risks impact host ecology. Many future avenues of work remain that would complement the ideas presented in this manuscript. For instance, individual variation owing to acquired immunity or plastic behavioural responses may impact how a potential host interacts with the landscape of disgust [117] and should be investigated alongside repeatable host behavioural defences (e.g. ‘hygienic personalities’ [118]). Exploring whether some of the landscape of disgust concepts may be applied to other parasitism models that do not have classic host–parasite dynamics would also be valuable (e.g. individuals use visual cues to detect brood parasitism [119]). Little work has explored how parasites may interact with, or compensate for, the landscape of disgust. Finally, because ecology and evolution can have reciprocal effects [23], we also suggest investigations that explore how these two processes could interact to influence the landscape of disgust, particularly in natural systems. Clearly, many outstanding questions regarding the landscape of disgust framework remain. We focus our discussion above on habitat selection and evolutionary theories given that they are the focus of the current paper, although a longer-term goal for the landscape of disgust should include integration with other frameworks and theories to create a more holistic—and therefore even more powerful— framework.

## Data Availability

This article has no additional data.

## References

[1] Hart BL. 1990 Behavioral adaptations to pathogens and parasites: five strategies. Neurosci. & Biobehav. Rev. **14**, 273–294. (10.1016/S0149-7634(05)80038-7) 2234607

[2] Lima SL, Dill LM. 1990 Behavioral decisions made under the risk of predation: a review and prospectus. Can. J. Zool. **68**, 619–640. (10.1139/z90-092)

[3] Anderson RM, May RM. 1982 Coevolution of hosts and parasites. Parasitology **85**, 411–426. (10.1017/S0031182000055360) 6755367

[4] Weinstein SB, Buck JC, Young HS. 2018 A landscape of disgust. Science **359**, 1213–1214. (10.1126/science.aas8694) 29590062

[5] Buck JC, Weinstein SB, Young HS. 2018 Ecological and evolutionary consequences of parasite avoidance. Trends Ecol. Evol. **33**, 619–632. (10.1016/j.tree.2018.05.001) 29807838

[6] Lopes PC, French SS, Woodhams DC, Binning SA. 2022 *infection avoidance behaviors across vertebrate taxa: patterns, processes, and future directions*. In Animal behavior and parasitism (eds V Ezenwa, SM Altizer, R Hall), pp. 237–256. Oxford, UK: Oxford University Press. (10.1093/oso/9780192895561.003.0014)

[7] Parker BJ, Barribeau SM, Laughton AM, de Roode JC, Gerardo NM. 2011 Non-immunological defense in an evolutionary framework. Trends Ecol. Evol. **26**, 242–248. (10.1016/j.tree.2011.02.005) 21435735

[8] Hart BL. 2011 Behavioural defences in animals against pathogens and parasites: parallels with the pillars of medicine in humans. Phil. Trans. R. Soc. B **366**, 3406–3417. (10.1098/rstb.2011.0092) 22042917 PMC3189355

[9] de Roode JC, Lefèvre T. 2012 Behavioral immunity in insects. Insects **3**, 789–820. (10.3390/insects3030789) 26466629 PMC4553590

[10] Stockmaier S, Ulrich Y, Albery GF, Cremer S, Lopes PC. 2023 Behavioural defences against parasites across host social structures. Funct. Ecol. **37**, 809–820. (10.1111/1365-2435.14310)

[11] Gibson AK, Amoroso CR. 2022 Evolution and ecology of parasite avoidance. Annu. Rev. Ecol. Evol. Syst. **53**, 47–67. (10.1146/annurev-ecolsys-102220-020636) 36479162 PMC9724790

[12] Behringer DC, Karvonen A, Bojko J. 2018 Parasite avoidance behaviours in aquatic environments. Phil. Trans. R. Soc. B **373**, 20170202. (10.1098/rstb.2017.0202) 29866915 PMC6000143

[13] Brown JS, Laundre JW, Gurung M. 1999 The ecology of fear: optimal foraging, game theory, and trophic interactions. J. Mammal. **80**, 385–399. (10.2307/1383287)

[14] Doherty JF, Ruehle B. 2020 An integrated landscape of fear and disgust: the evolution of avoidance behaviors amidst a myriad of natural enemies. Front. Ecol. Evol. **8**, 564343. (10.3389/fevo.2020.564343)

[15] Sarabian C, Wilkinson A, Sigaud M, Kano F, Tobajas J, Darmaillacq AS, Kalema-Zikusoka G, Plotnik JM, MacIntosh AJJ. 2023 Disgust in animals and the application of disease avoidance to wildlife management and conservation. J. Anim. Ecol. **92**, 1489–1508. (10.1111/1365-2656.13903) 36914973

[16] Daversa DR, Hechinger RF, Madin E, Fenton A, Dell AI, Ritchie EG, Rohr J, Rudolf VHW, Lafferty KD. 2021 Broadening the ecology of fear: non-lethal effects arise from diverse responses to predation and parasitism. Proc. R. Soc. B **288**, 20202966. (10.1098/rspb.2020.2966) PMC7935051 33622122

[17] Peckarsky BL *et al*. 2008 Revisiting the classics: considering nonconsumptive effects in textbook examples of predator–prey interactions. Ecology **89**, 2416–2425. (10.1890/07-1131.1) 18831163

[18] Koprivnikar J, Rochette A, Forbes MR. 2021 Risk-induced trait responses and non-consumptive effects in plants and animals in response to their invertebrate herbivore and parasite natural enemies. Front. Ecol. Evol. **9**, 667030. (10.3389/fevo.2021.667030)

[19] Grenfell BT, Dobson AP. 1995 Ecology of infectious diseases in natural populations. Cambridge, UK: Cambridge University Press.

[20] Hasik AZ, Siepielski AM. 2022 Parasitism shapes selection by drastically reducing host fitness and increasing host fitness variation. Biol. Lett. **18**, 20220323. (10.1098/rsbl.2022.0323) 36321430 PMC9627441

[21] Roy BA, Kirchner JW. 2000 Evolutionary dynamics of pathogen resistance and tolerance. Evolution **54**, 51–63. (10.1111/j.0014-3820.2000.tb00007.x) 10937183

[22] Morris DW. 1987 Ecological scale and habitat use. Ecology **68**, 362–369. (10.2307/1939267)

[23] Hendry AP. Eco-evolutionary dynamics. Princeton, NJ: Princeton University Press. (10.1515/9781400883080)

[24] Trail D. 1980 Behavioral interactions between parasites and hosts: host suicide and the evolution of complex life cycles. Am. Nat. **116**, 77–91. (10.1086/283612)

[25] Kavaliers M, Choleris E, Agmo A, Pfaff DW. 2004 Olfactory-mediated parasite recognition and avoidance: linking genes to behavior. Horm. Behav. **46**, 272–283. (10.1016/j.yhbeh.2004.03.005) 15325228

[26] Kavaliers M, Choleris E. 2018 The role of social cognition in parasite and pathogen avoidance. Phil. Trans. R. Soc. B **373**, 20170206. (10.1098/rstb.2017.0206) 29866919 PMC6000139

[27] Klemme I, Hyvärinen P, Karvonen A. 2020 Negative associations between parasite avoidance, resistance and tolerance predict host health in salmonid fish populations. Proc. R. Soc. B **287**, 20200388. (10.1098/rspb.2020.0388) PMC7211438 32315591

[28] Keymer A, Crompton DWT, Sahakian BJ. 1983 Parasite-induced learned taste aversion involving Nippostrongylus in rats. Parasitology **86**, 455–460. (10.1017/s0031182000050642) 6877871

[29] Hart BL, Hart LA. 2018 How mammals stay healthy in nature: the evolution of behaviours to avoid parasites and pathogens. Phil. Trans. R. Soc. B **373**, 20170205. (10.1098/rstb.2017.0205) 29866918 PMC6000140

[30] Vinkler M *et al*. 2023 Understanding the evolution of immune genes in jawed vertebrates. J. Evol. Biol. **36**, 847–873. (10.1111/jeb.14181) 37255207 PMC10247546

[31] Loudon JE, Sauther ML, Fish KD, Hunter-Ishikawa M, Ibrahim YJ. 2006 One reserve, three primates: applying a holistic approach to understand the interconnections among ring-tailed lemurs (Lemur catta), Verreaux’s sifaka (Propithecus verreauxi), and humans (Homo sapiens) at Beza Mahafaly Special Reserve, Madagascar. Ecol. Environ. Anthropol. **2**, 54–74.

[32] Bustnes JO, Galaktionov KV. 2004 Evidence of a state-dependent trade-off between energy intake and parasite avoidance in Steller’s eiders. Can. J. Zool. **82**, 1566–1571. (10.1139/z04-139)

[33] Hutchings MR, Kyriazakis I, Anderson DH, Gordon IJ, Coop RL. 1998 Behavioural strategies used by parasitized and non-parasitized sheep to avoid ingestion of gastro-intestinal nematodes associated with faeces. Anim. Sci. **67**, 97–106. (10.1017/S1357729800009838)

[34] Selbach C, Marchant L, Mouritsen KN. 2022 Mussel memory: can bivalves learn to fear parasites? R. Soc. Open Sci. **9**, 211774. (10.1098/rsos.211774) 35116166 PMC8790352

[35] Tacey J, Class B, Delmé C, Powell D, Frère CH. 2023 Impacts of fungal disease on dyadic social interactions in a wild agamid lizard. Anim. Behav. **200**, 125–136. (10.1016/j.anbehav.2023.04.002)

[36] Sarabian C, Belais R, MacIntosh AJJ. 2021 Avoidance of contaminated food correlates with low protozoan infection in bonobos. Front. Ecol. Evol. **9**, 651159. (10.3389/fevo.2021.651159)

[37] Jordan LA, Ryan MJ. 2015 The sensory ecology of adaptive landscapes. Biol. Lett. **11**, 20141054. (10.1098/rsbl.2014.1054) 26018831 PMC4455732

[38] Elith J, Leathwick JR. 2009 Species distribution models: ecological explanation and prediction across space and time. Annu. Rev. Ecol. Evol. Syst. **40**, 677–697. (10.1146/annurev.ecolsys.110308.120159)

[39] Northrup JM, Vander Wal E, Bonar M, Fieberg J, Laforge MP, Leclerc M, Prokopenko CM, Gerber BD. 2022 Conceptual and methodological advances in habitat-selection modeling: guidelines for ecology and evolution. Ecol. Appl. **32**, e02470. (10.1002/eap.2470) 34626518 PMC9285351

[40] Dallas T, Gehman AM, Aguirre AA, Budischak SA, Drake JM, Farrell MJ, Ghai R, Huang S, Morales‐Castilla I. 2019 Contrasting latitudinal gradients of body size in helminth parasites and their hosts. Glob. Ecol. Biogeogr. **28**, 804–813. (10.1111/geb.12894)

[41] Norberg A *et al*. 2019 A comprehensive evaluation of predictive performance of 33 species distribution models at species and community levels. Ecol. Monogr. **89**, e01370. (10.1002/ecm.1370)

[42] Malishev M, Kramer-Schadt S. 2021 Movement, models, and metabolism: individual-based energy budget models as next-generation extensions for predicting animal movement outcomes across scales. Ecol. Modell. **441**, 109413. (10.1016/j.ecolmodel.2020.109413)

[43] Ferraro KM, Schmitz OJ, McCary MA. 2022 Effects of ungulate density and sociality on landscape heterogeneity: a mechanistic modeling approach. Ecography **2022**. (10.1111/ecog.06039)

[44] Visscher PM, Hill WG, Wray NR. 2008 Heritability in the genomics era concepts and misconceptions. Nat. Rev. Genet. **9**, 255–266. (10.1038/nrg2322) 18319743

[45] Lande R. 1979 Quantitative genetic analysis of multivariate evolution, applied to brain: body size allometry. Evolution **33**, 402. (10.2307/2407630) 28568194

[46] Lande R, Arnold SJ. 1983 The measurement of selection on correlated characters. Evolution **37**, 1210. (10.2307/2408842) 28556011

[47] Matthiopoulos J, Fieberg J, Aarts G. Species-habitat associations: spatial data, predictive models, and ecological insights. Minneapolis, MN: University of Minnesota Libraries Publishing. (10.24926/2020.081320)

[48] Morris DW. 2011 Adaptation and habitat selection in the eco-evolutionary process. Proc. R. Soc. B **278**, 2401–2411. (10.1098/rspb.2011.0604) PMC3125633 21613295

[49] Northrup JM, Vander Wal E, Bonar M, Fieberg J, Laforge MP, Leclerc M, Prokopenko CM, Gerber BD. 2022 Conceptual and methodological advances in habitat‐selection modeling: guidelines for ecology and evolution. Ecol. Appl. **32**, e02470. (10.1002/eap.2470) 34626518 PMC9285351

[50] Morris DW. 2003 Toward an ecological synthesis: a case for habitat selection. Oecologia **136**, 1–13. (10.1007/s00442-003-1241-4) 12690550

[51] Fretwell SD, Lucas HLJ. 1969 On territorial behavior and other factors influencing habitat distribution in birds. Acta Biotheor. **19**, 16–36. (10.1007/BF01601953)

[52] Hochachka WM, Dhondt AA. 2000 Density-dependent decline of host abundance resulting from a new infectious disease. Proc. Natl Acad. Sci. USA **97**, 5303–5306. (10.1073/pnas.080551197) 10792031 PMC25823

[53] May RM, Anderson RM. 1979 Population biology of infectious diseases: part II. Nature **280**, 455–461. (10.1038/280455a0) 460424

[54] Patterson JEH, Ruckstuhl KE. 2013 Parasite infection and host group size: a meta-analytical review. Parasitology **140**, 803–813. (10.1017/S0031182012002259) 23425516 PMC3638372

[55] Cote IM, Poulinb R. 1995 Parasitism and group size in social animals: a meta-analysis. Behav. Ecol. **6**, 159–165. (10.1093/beheco/6.2.159)

[56] Mooring MS, Hart BL. 1992 Animal grouping for protection from parasites: selfish herd and encounter-dilution effects. Behaviour **123**, 173–193. (10.1163/156853992X00011)

[57] Albery GF, Newman C, Ross JB, MacDonald DW, Bansal S, Buesching C. 2020 Negative density-dependent parasitism in a group-living carnivore. Proc. R. Soc. B **287**, 20202655. (10.1098/rspb.2020.2655) PMC7779509 33323092

[58] Stewart Merrill TE, Cáceres CE, Gray S, Laird VR, Schnitzler ZT, Buck JC. 2022 Timescale reverses the relationship between host density and infection risk. Proc. R. Soc. B **289**, 20221106. (10.1098/rspb.2022.1106) PMC9346366 35919996

[59] Titcomb G, Mantas JN, Hulke J, Rodriguez I, Branch D, Young H. 2021 Water sources aggregate parasites with increasing effects in more arid conditions. Nat. Commun. **12**, 7066. (10.1038/s41467-021-27352-y) 34862389 PMC8642388

[60] Escobar LE, Moen R, Craft ME, VanderWaal KL. 2019 Mapping parasite transmission risk from white-tailed deer to a declining moose population. Eur. J. Wildl. Res. **65**, 60. (10.1007/s10344-019-1297-z)

[61] Ward AJW, Duff AJ, Krause J, Barber I. 2005 Shoaling behaviour of sticklebacks infected with the microsporidian parasite, Glugea anomala. Environ. Biol. Fishes **72**, 155–160. (10.1007/s10641-004-9078-1)

[62] Turner JW, Prokopenko CM, Kingdon KA, Dupont DLJ, Zabihi-Seissan S, Vander Wal E. 2023 Death comes for us all: relating movement-integrated habitat selection and social behavior to human-associated and disease-related mortality among gray wolves. Oecologia **202**, 685–697. (10.1007/s00442-023-05426-6) 37515598

[63] Croft DP, Edenbrow M, Darden SK, Ramnarine IW, van Oosterhout C, Cable J. 2011 Effect of gyrodactylid ectoparasites on host behaviour and social network structure in guppies Poecilia reticulata. Behav. Ecol. Sociobiol. **65**, 2219–2227. (10.1007/s00265-011-1230-2)

[64] Webber QMR, Albery GF, Farine DR, Pinter‐Wollman N, Sharma N, Spiegel O, Vander Wal E, Manlove K. 2023 Behavioural ecology at the spatial–social interface. Biol. Rev. **98**, 868–886. (10.1111/brv.12934) 36691262

[65] Antonovics J *et al*. 2017 The evolution of transmission mode. Phil. Trans. R. Soc. B **372**, 20160083. (10.1098/rstb.2016.0083) 28289251 PMC5352810

[66] Rifkin JL, Nunn CL, Garamszegi LZ. 2012 Do animals living in larger groups experience greater parasitism? A meta-analysis. Am. Nat. **180**, 70–82. (10.1086/666081) 22673652

[67] Wilson SN, Sindi SS, Brooks HZ, Hohn ME, Price CR, Radunskaya AE, Williams ND, Fefferman NH. 2020 How emergent social patterns in allogrooming combat parasitic infections. Front. Ecol. Evol. **8**, 54. (10.3389/fevo.2020.00054)

[68] Gilbert KA. 1997 Red howling monkey use of specific defecation sites as a parasite avoidance strategy. Anim. Behav. **54**, 451–455. (10.1006/anbe.1996.0439) 9268477

[69] Hausfater G, Meade BJ. 1982 Alternation of sleeping groves by yellow baboons (Papio cynocephalus) as a strategy for parasite avoidance. Primates. **23**, 287–297. (10.1007/BF02381167)

[70] Reckardt K, Kerth G. 2007 Roost selection and roost switching of female Bechstein’s bats (Myotis bechsteinii) as a strategy of parasite avoidance. Oecologia **154**, 581–588. (10.1007/s00442-007-0843-7) 17805579

[71] Bailey DW, Gross JE, Laca EA, Rittenhouse LR, Coughenour MB, Swift DM, Sims PL. 1996 Mechanisms that result in large herbivore grazing distribution patterns. J. Range Manag. **49**, 386. (10.2307/4002919)

[72] Mayor SJ, Schneider DC, Schaefer JA, Mahoney SP. 2009 Habitat selection at multiple scales. Écoscience. **16**, 238–247. (10.2980/16-2-3238)

[73] Johnson DH. 1980 The comparison of usage and availability measurements for evaluating resource preference. Ecology **61**, 65–71. (10.2307/1937156)

[74] Gonzálvez M, Martínez-Carrasco C, Sánchez-Zapata JA, Moleón M. 2021 Smart carnivores think twice: red fox delays scavenging on conspecific carcasses to reduce parasite risk. Appl. Anim. Behav. Sci. **243**, 105462. (10.1016/j.applanim.2021.105462) 34602687 PMC8464160

[75] Gonzálvez M, Martínez-Carrasco C, Moleón M. 2021 Understanding potential implications for non-trophic parasite transmission based on vertebrate behavior at mesocarnivore carcass sites. Vet. Res. Commun. **45**, 261–275. (10.1007/s11259-021-09806-2) 34176034 PMC8235911

[76] Moisés G, Martínez-Carrasco C, Marcos M. 2021 Exploring vertebrate behavior at mesocarnivore carcass sites: implications for non-trophic parasite transmission. Research Square. (10.21203/rs.3.rs-488230/v1) PMC8235911 34176034

[77] Weinstein SB, Moura CW, Mendez JF, Lafferty KD. 2018 Fear of feces? Tradeoffs between disease risk and foraging drive animal activity around raccoon latrines. Oikos **127**, 927–934. (10.1111/oik.04866)

[78] Sarabian C, Ngoubangoye B, MacIntosh AJJ. 2017 Avoidance of biological contaminants through sight, smell and touch in chimpanzees. R. Soc. Open Sci. **4**, 170968. (10.1098/rsos.170968) 29291090 PMC5717664

[79] Sarabian C, Belais R, MacIntosh AJJ. 2018 Feeding decisions under contamination risk in bonobos. Phil. Trans. R. Soc. B **373**, 20170195. (10.1098/rstb.2017.0195) 29866924 PMC6000142

[80] Koprivnikar J, Penalva L. 2015 Lesser of two evils? Foraging choices in response to threats of predation and parasitism. PLoS One **10**, e0116569. (10.1371/journal.pone.0116569) 25635765 PMC4312073

[81] Sharp JG, Garnick S, Elgar MA, Coulson G. 2015 Parasite and predator risk assessment: nuanced use of olfactory cues. Proc. R. Soc. B **282**, 20151941. (10.1098/rspb.2015.1941) PMC4633877 26468246

[82] Keith SA, Drury JP, McGill BJ, Grether GF. 2023 Macrobehaviour: behavioural variation across space, time, and taxa. Trends Ecol. Evol. **38**, 1177–1188. (10.1016/j.tree.2023.08.007) 37661519

[83] Short EE, Caminade C, Thomas BN. 2017 Climate change contribution to the emergence or re-emergence of parasitic diseases. Infect. Dis. **10**, 1178633617732296. (10.1177/1178633617732296) PMC5755797 29317829

[84] Martin LB, Hopkins WA, Mydlarz LD, Rohr JR. 2010 The effects of anthropogenic global changes on immune functions and disease resistance. Ann. N. Y. Acad. Sci. **1195**, 129–148. (10.1111/j.1749-6632.2010.05454.x) 20536821

[85] Altizer S, Bartel R, Han BA. 2011 Animal migration and infectious disease risk. Science **331**, 296–302. (10.1126/science.1194694) 21252339

[86] Busch DS, Robinson WD, Robinson TR, Wingfield JC. 2011 Influence of proximity to a geographical range limit on the physiology of a tropical bird. J. Anim. Ecol. **80**, 640–649. (10.1111/j.1365-2656.2010.01791.x) 21219328

[87] Lomolino MV, Rosenzweig ML. 1996 Species diversity in space and time. J. Wildl. Manage. **60**, 971. (10.2307/3802400)

[88] Huang ZYX, VAN Langevelde F, Estrada-Peña A, Suzán G, DE Boer WF. 2016 The diversity-disease relationship: evidence for and criticisms of the dilution effect. Parasitology **143**, 1075–1086. (10.1017/S0031182016000536) 27041655

[89] Robertson SL, Hamilton IM. 2012 Habitat selection under the risk of infectious disease. Evol. Ecol. Res. **14**, 51–72.

[90] Mierzejewski MK, Horn CJ, Luong LT. 2019 Ecology of fear: environment-dependent parasite avoidance among ovipositing Drosophila Parasitology **146**, 1564–1570. (10.1017/S0031182019000854) 31234951

[91] Hutchings MR, Milner JM, Gordon IJ, Kyriazakis I, Jackson F. 2002 Grazing decisions of Soay sheep, Ovis aries, on St Kilda: a consequence of parasite distribution? Oikos **96**, 235–244. (10.1034/j.1600-0706.2002.960205.x)

[92] Palmer MS, Gaynor KM, Becker JA, Abraham JO, Mumma MA, Pringle RM. 2022 Dynamic landscapes of fear: understanding spatiotemporal risk. Trends Ecol. Evol. **37**, 911–925. (10.1016/j.tree.2022.06.007) 35817684

[93] Whitlaw HA, Lankester MW. 1994 The co-occurrence of moose, white-tailed deer, and Parelaphostrongylus tenuis in ontario. Can. J. Zool. **72**, 819–825. (10.1139/z94-111)

[94] Poulin R, Bennett J, de Angeli Dutra D, Doherty JF, Filion A, Park E, Ruehle B. 2020 Evolutionary signature of ancient parasite pressures, or the ghost of parasitism past. Front. Ecol. Evol. **8**, 195. (10.3389/fevo.2020.00195)

[95] Zuk M, Rotenberry JT, Tinghitella RM. 2006 Silent night: adaptive disappearance of a sexual signal in a parasitized population of field crickets. Biol. Lett. **2**, 521–524. (10.1098/rsbl.2006.0539) 17148278 PMC1834006

[96] Ghalambor CK, McKAY JK, Carroll SP, Reznick DN. 2007 Adaptive versus non‐adaptive phenotypic plasticity and the potential for contemporary adaptation in new environments. Funct. Ecol. **21**, 394–407. (10.1111/j.1365-2435.2007.01283.x)

[97] Sarabian C, Curtis V, McMullan R. 2018 Evolution of pathogen and parasite avoidance behaviours. Phil. Trans. R. Soc. B **373**, 20170256. (10.1098/rstb.2017.0256) 29866923 PMC6000144

[98] Boots M, Bowers RG. 1999 Three mechanisms of host resistance to microparasites-avoidance, recovery and tolerance-show different evolutionary dynamics. J. Theor. Biol. **201**, 13–23. (10.1006/jtbi.1999.1009) 10534432

[99] Zylberberg M, Klasing KC, Hahn TP. 2013 House finches (Carpodacus mexicanus) balance investment in behavioural and immunological defences against pathogens. Biol. Lett. **9**, 20120856. (10.1098/rsbl.2012.0856) 23134781 PMC3565497

[100] Amoroso CR, Antonovics J. 2020 Evolution of behavioural resistance in host-pathogen systems. Biol. Lett. **16**, 20200508. (10.1098/rsbl.2020.0508) 32933405 PMC7532708

[101] Hamilton WD, Zuk M. 1982 Heritable true fitness and bright birds: a role for parasites? Science **218**, 384–387. (10.1126/science.7123238) 7123238

[102] Møller AP, Nielsen JT. 1997 Differential predation cost of a secondary sexual character: sparrowhawk predation on barn swallows. Anim. Behav. **54**, 1545–1551. (10.1006/anbe.1997.9998) 9794779

[103] Balenger SL, Zuk M. 2014 Testing the Hamilton-Zuk hypothesis: past, present, and future. Integr. Comp. Biol. **54**, 601–613. (10.1093/icb/icu059) 24876194

[104] Joye P, Kawecki TJ. 2019 Sexual selection favours good or bad genes for pathogen resistance depending on males’ pathogen exposure. Proc. R. Soc. B **286**, 20190226. (10.1098/rspb.2019.0226) PMC6532500 31064300

[105] Heckley AM, de Lira JJPR, Hendry AP, Pérez-Jvostov F. 2022 How might Gyrodactylus parasitism modify trade-offs between female preference and susceptibility of males to predation in Trinidadian guppies? Int. J. Parasitol. **52**, 459–467. (10.1016/j.ijpara.2022.01.006) 35331715

[106] Frankham R. 2010 Challenges and opportunities of genetic approaches to biological conservation. Biol. Conserv. **143**, 1919–1927. (10.1016/j.biocon.2010.05.011)

[107] Kaltz O, Shykoff JA. 1998 Local adaptation in host–parasite systems. Heredity **81**, 361–370. (10.1046/j.1365-2540.1998.00435.x)

[108] Chabas H, van Houte S, Høyland-Kroghsbo NM, Buckling A, Westra ER. 2016 Immigration of susceptible hosts triggers the evolution of alternative parasite defence strategies. Proc. R. Soc. B **283**, 20160721. (10.1098/rspb.2016.0721) PMC5013786 27581884

[109] Moro A, Blacquière T, Panziera D, Dietemann V, Neumann P. 2021 Host–parasite co-evolution in real-time: changes in honey bee resistance mechanisms and mite reproductive strategies. Insects **12**, 120. (10.3390/insects12020120) 33572966 PMC7911685

[110] Gandon S, Michalakis Y. 2000 Evolution of parasite virulence against qualitative or quantitative host resistance. Proc. R. Soc. B **267**, 985–990. (10.1098/rspb.2000.1100) PMC1690632 10874747

[111] He X *et al*. 2020 Temporal dynamics in viral shedding and transmissibility of COVID-19. Nat. Med. **26**, 672–675. (10.1038/s41591-020-0869-5) 32296168

[112] Rogalski MA, Gowler CD, Shaw CL, Hufbauer RA, Duffy MA. 2017 Human drivers of ecological and evolutionary dynamics in emerging and disappearing infectious disease systems. Phil. Trans. R. Soc. B **372**, 20160043. (10.1098/rstb.2016.0043) 27920388 PMC5182439

[113] Dunn AM. 2009 Parasites and biological invasions. Adv. Parasitol. **68**, 161–184. (10.1016/S0065-308X(08)00607-6) 19289194

[114] Vilcinskas A. 2015 Pathogens as biological weapons of invasive species. PLoS Pathog. **11**, e1004714. (10.1371/journal.ppat.1004714) 25856550 PMC4391917

[115] Rushton SP, Lurz PWW, Gurnell J, Fuller R. 2000 Modelling the spatial dynamics of parapoxvirus disease in red and grey squirrels: a possible cause of the decline in the red squirrel in the UK? J. Appl. Ecol. **37**, 997–1012. (10.1046/j.1365-2664.2000.00553.x)

[116] DeAngelis DL, Diaz SG. 2019 Decision-making in agent-based modeling: a current review and future prospectus. Front. Ecol. Evol. **6**, 237. (10.3389/fevo.2018.00237)

[117] Barron D, Gervasi S, Pruitt J, Martin L. 2015 Behavioral competence: how host behaviors can interact to influence parasite transmission risk. Curr. Opin. Behav. Sci. **6**, 35–40. (10.1016/j.cobeha.2015.08.002)

[118] Poirotte C, Kappeler PM. 2019 Hygienic personalities in wild grey mouse lemurs vary adaptively with sex. Proc. R. Soc. B **286**, 20190863. (10.1098/rspb.2019.0863) PMC6710582 31387505

[119] Spottiswoode CN, Stevens M. 2010 Visual modeling shows that avian host parents use multiple visual cues in rejecting parasitic eggs. Proc. Natl Acad. Sci. USA **107**, 8672–8676. (10.1073/pnas.0910486107) 20421497 PMC2889299

